# Global gridded multi-temporal datasets to support human population distribution modelling

**DOI:** 10.12688/gatesopenres.16363.1

**Published:** 2025-09-15

**Authors:** Dorothea Woods, Tom McKeen, Alexander Cunningham, Rhorom Priyatikanto, Andrew J. Tatem, Alessandro Sorichetta, Maksym Bondarenko

**Affiliations:** 1 University of Southampton, Southampton, England, UK; 2 Università degli Studi di Milano Scienze della Terra, Milan, Lombardy, Italy

**Keywords:** spatial demography; geospatial covariates; high-resolution gridded data; human population; subnational; global; spatial dataset; multi-temporal

## Abstract

Population distributions across countries and regions exhibit significant spatial and temporal variability. This variation highlights the need for high-resolution, small-area demographic data to address the challenges posed by shifting population dynamics, urbanization, and migration. Small area population modelling, particularly the production of gridded population estimates, has advanced rapidly over the past decade. Gridded population estimates rely heavily on the availability of detailed geospatial ancillary datasets to capture, inform and explain the variabilities in population densities and distributions at small area scales, enabling the disaggregation from areal unit-based counts. Here we describe an extensive geospatial collection of annual, high resolution, spatio-temporally harmonised, global datasets aimed at driving improvements in mapping small area population density variation. This article presents the spatio-temporal harmonisation process that results in an open access repository of 73 individual gridded datasets addressing topography, climate, nighttime lights, land cover, inland water, infrastructure, protected areas as well as the built-up environment on a global level at a spatial resolution of 3 arc-seconds (approximately 100 metres). Datasets are available as annual time series from 2015 up to and including at least 2020, and as recent as 2023 where source datasets allow. Such datasets not only support population modelling but also applications across environmental, economic, and health sectors, supporting informed policy-making and resource allocation for sustainable development.

## Background & Summary

The global population is estimated to have reached 8 billion in November 2022 and is projected to peak at 10.3 billion by the mid-2080s, largely due to increased numbers of women of reproductive age despite near-replacement fertility rates. ^1^ After this peak, a gradual decline is expected, with some countries already seeing declines. While 63 countries (28% of the global population) have peaked, and 48 others (10%) are expected to peak by 2054, the remaining 126 countries (62%) may continue growing until later in the century, driven by immigration and high fertility, especially in sub-Saharan Africa where populations could double by mid-century. ^1^ At the subnational level, the changing demographic landscape across space and time is even more complex with fertility and mortality rates differing between urban and rural settings, ^2, 3^ the continuing rapid processes of urbanisation especially in the Global South, ^4, 5^ but also the growing phenomena of counter-urbanisation where people relocate from urban centres to rural areas. ^6, 7^ Events like conflict and natural disaster can displace entire cohorts within short timescales and have the potential to impact the subnational distribution of populations substantially. ^8– 10^ These variations in population densities at small area scales drives the need for consistent, multi-temporal demographic data to allow detailed insights at a granular level. Such data can support the detection of demographic trends, the effective allocation of resources, the assessment of impacts of events and the design of successful interventions, as well as the tracking of development goals and ultimately support policy- and decision-making to drive sustainable development.

The field of small area population modelling has developed rapidly over the last decade and complements more traditional demographic data like the national population and housing censuses, household surveys and administrative records. To maintain confidentiality, traditional data are typically only released publicly after spatial aggregation into larger geographical units. This loss of spatial detail in itself brings problems which are intensified by the fact that published demographic data are strictly dependent on the chosen boundaries they are released with. These are often of administrative nature and do not match boundaries of future analysis, for example practical applications like mapping of school catchments or watersheds straddling across administrative boundaries. International efforts are undertaken to promote the use of harmonised grids for demographic data distribution instead ^11^ but to-date most demographic data is still published as population counts linked to geographic areas of varying sizes. Small area gridded population estimates overcome this problem by disaggregating traditionally observed population counts to small equally sized uniform grid cells (pixels), often of 100 metres or 1 kilometre resolution, which can be flexibly aggregated to any desired boundaries. As such the disaggregation of population estimates from large administrative areas to small scale grid cells, referred to as ‘top-down’ disaggregation method, ^12^ forms an invaluable tool to enable the wider use of demographic data.

Various methods are used to produce global gridded population data. These range from simple areal-weighting approaches where population counts are evenly distributed across areal units ^13^ as in the Gridded Population of the World (GPW) v4 dataset, ^14^ to statistical models leveraging multi-temporal geospatial datasets on settlements and other related features to disaggregate population counts from larger areal units. Binary dasymetric models use masking techniques to restrict population disaggregation into urban extents (the method used in the Global Rural Urban Mapping Project (GRUMP), ^15^ while more complex models rely on the presence or characteristics of individual buildings and built-up areas (examples include High Resolution Population Density Maps (HRPDM), ^16^ Global Human Settlement Layer Population (GHS-POP), ^17^ and POPulation from COaRse census Numbers (POPCORN). ^18^ Moreover, dasymetric models integrate multiple ancillary datasets to refine population distribution (e.g. LandScan) ^19^ and WorldPop’s population models. ^20– 22^ In addition to these top-down techniques that disaggregate national census data and projections into small-area grid cells, bottom-up approaches also employ ancillary geospatial datasets to generate population estimates at fine spatial scales, particularly in regions where census data are missing, unreliable or incomplete due to factors such as inaccessibility or conflict. ^12^ Such models rely on population enumeration data from sample areas, such as from micro-censuses, incomplete censuses or household survey listings, and combine these data with other geospatial datasets to construct models that are used to predict population numbers in unsampled areas, thereby achieving comprehensive spatial coverage. ^23– 25^

The different types of models outlined above highlights the extensive use of ancillary geospatial datasets in generating accurate population estimates in both top-down and bottom-up approaches. While spatial population distribution is influenced by a variety of factors, universal patterns exist. ^26^ To provide insight, ancillary datasets need to be closely associated to population presence such as location of residential buildings, population absence like waterbodies, or variations in population densities, which for example can be correlated with the amount of artificial light emitted at night or the proximity to transportation networks. A study conducted across 32 countries revealed that certain geographical features consistently drive population distribution, regardless of local contexts. ^26^ These features include geospatial datasets associated with the built-up environment, ecology, and topography, which collectively accounted for much of the variability in population distributions observed, even at fine spatial resolutions. In recent years, the importance of nighttime lights and datasets depicting the built-up environment has been increasingly recognised in top-down population models. ^27– 30^ Following these findings, we here collate a collection of global geospatial layers depicting topography, climate, nighttime lights, land cover, protected areas, infrastructure and the built-up environment and provide them in a gridded format.

It is paramount for ancillary geospatial datasets, from here-on referred to as covariates, to be spatially co-registered to the target grid. In recent years, repositories of standardised gridded geospatial covariates have been developed using open source geospatial software and geographical information systems (GIS). This development has been driven by the growing need for multi-temporal covariates to monitor population and environmental changes over time, as well as the availability of new source datasets that facilitate such analyses. Examples of such datasets - produced as tiles, by country, or as global mosaics at spatial resolutions of 3 and 30 arc-seconds (i.e. respectively approximately 100 metres and 1 kilometre at the Equator), include Lloyd, et al., ^31^ and Lloyd, et al. ^32^

In this paper we describe the spatio-temporal harmonisation process used to create a new open access repository of near global standardised gridded geospatial covariates. The methods include advances over those employed to develop previous repositories of this type. There are three marked differences: 1) incorporation of new and/or higher spatial resolution source datasets, most notably the expansion to seven source datasets describing the built-up environment, one additional infrastructure source dataset, a new high resolution inland water mask source dataset as well as new climatic and topographic datasets; 2) improved processing algorithms describing the built-up environment comprehensively through combination of multiple datasets, providing a series of seven inland water masks based on different thresholds of water coverage, and new outlier removal algorithm for nighttime lights; and 3) focussing on an annual time series for the recent time frame from 2015 up to and including at least 2020, and as recent as 2023 where source datasets allow.

The repository includes 73 datasets pertaining to building counts, building characteristics, building densities, built surface, built volume, water coverage, nighttime lights, topography, climate, infrastructure, road densities, as well as several distance-to datasets, which detail the distance to the closest road, multiple land cover classes and protected areas.

Production of the collection of covariates described here has been motivated by the production of a new global time series of high resolution age- and sex-structured population estimates by WorldPop. These new population estimates are intended to expand accessibility and usability of demographic dataset for the global public good. Usage of the covariates described in this paper is not limited to population modelling and can equally support a broad range of applications.

## Methods

**Figure 1.**
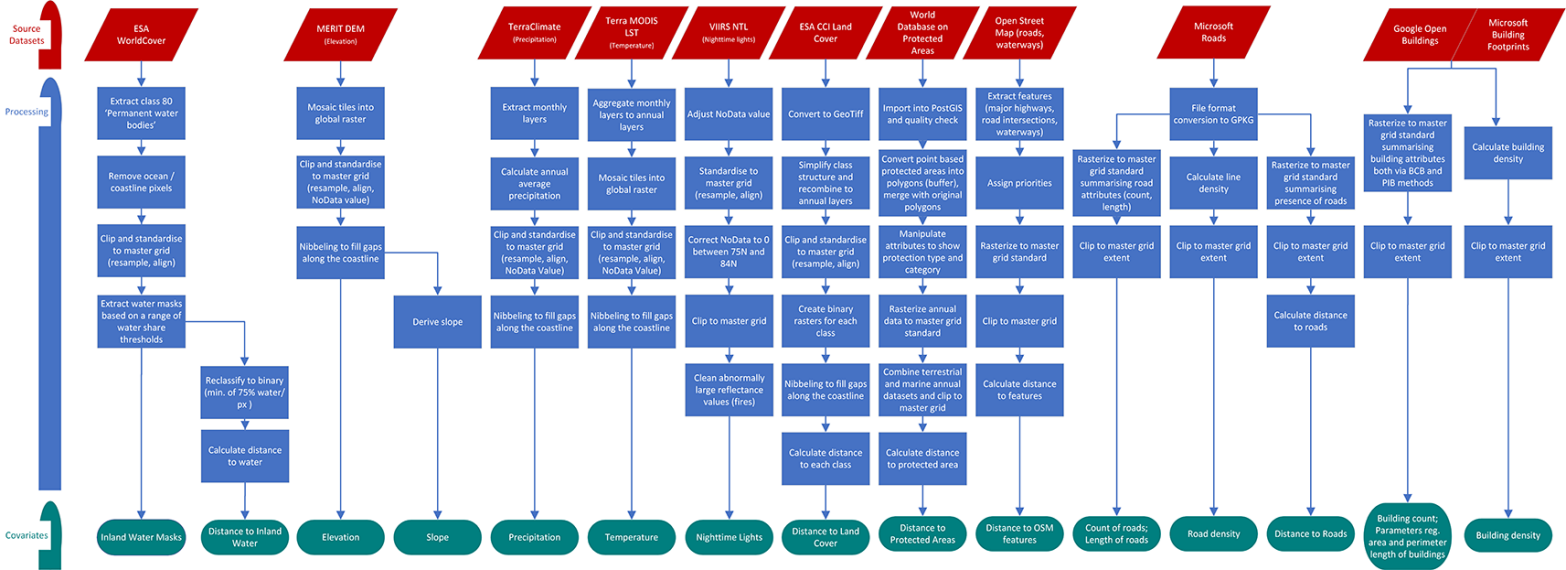
Flowchart of the spatio-temporal harmonisation process for all covariates (except covariates related to the built-up environment).

**Figure 2.**
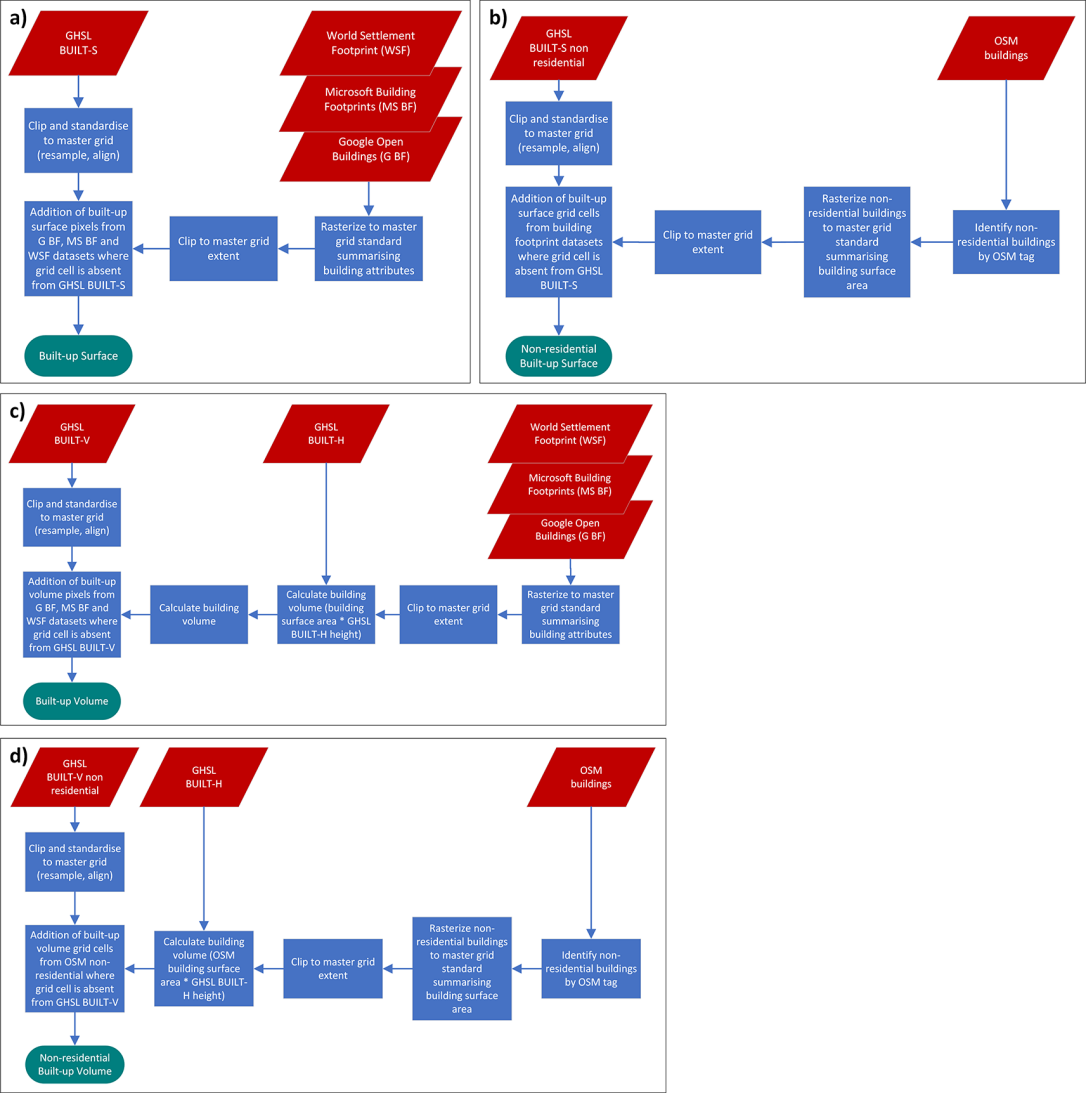
Flowchart of the spatio-temporal harmonisation process for the built-up environment. Part a), b), c), d) show the source datasets involved and the processing steps for the production of the covariates ‘built-up surface’, ‘non-residential built-up surface’, ‘built-up volume’ and ‘non-residential built-up volume’, respectively.

The following sections describe i) software, ii) selection of source datasets, iii) construction of a global master grid to co-register data to, and iv) the spatio-temporal covariate harmonisation process itself. The corresponding scripts describing the GIS workflows are openly accessible (see section Code Availability) and the resulting collection of datasets is available under one single citation (see section Data Availability). Figure 1 and Figure 2 show the spatio-temporal harmonisation process used to create an open access repository of gridded geospatial covariates.

### Processing software

The process to spatially harmonise covariates is predominantly carried out with open source geospatial software within a Windows 10 environment. The majority of processing utilises the geospatial library GDAL 3.6 ^33^ via the OSGeo4W Shell and batch scripts. For data exploration and visualisation the open source desktop GIS applications QGIS 3.22 ^34^ is used. In the case of processing of WDPA data ArcGIS Pro 3.0.2 ^35^ is also employed (see section Spatio-temporal Harmonisation of Covariates, 6. Protected Areas). Occasionally, processing steps are too computationally intensive for a personal computer and require the use of a high performance cluster (HPC). The HPC used runs on a RedHat Linux operating system.

### Source datasets

Source datasets must fulfil multiple criteria to be considered suitable for inclusion in this harmonised set of covariates. Firstly, they must represent a theme known to correlate with population distribution or be considered a proxy for it. Population distribution is shaped by a complex interplay of economic, environmental, geographical, political, social and other factors. Nieves, et al. ^26^ analysed the drivers of population distribution and found a set of geographical features relating to the built-up environment, ecology and topography consistently explain the majority of variability in population distributions at fine spatial scales. In recent years, the importance of nighttime lights and datasets depicting the built-up environment has been increasingly recognised in top-down population models. ^27– 30^ Following these findings, we include geospatial layers depicting the built-up environment, nighttime lights, topography, climate, land cover, protected areas, and infrastructure.

Besides thematic relevance, source datasets must be open access, have near-global coverage and be of sufficient spatial and temporal resolution. Most datasets fulfilling these requirements will have been produced through satellite remote sensing techniques and thus are in a raster format. The World Database on Protected Areas (WDPA), ^36^ OpenStreetMap (OSM) data ^37^ and building footprints ^38, 39^ form the exception and are available as vector data. As we are interested in the relationship of covariates to population density and distribution, source datasets need to cover populated areas. With this in mind, it is acceptable to include datasets which do not cover the highest latitudes where there is little landmass with negligible population.

To predict population on an approximately 100 metres grid (3 arc-seconds or 0.00083333333 decimal degrees), it is ideal for source datasets to match or exceed the resolution of the target grid. 11 of the 16 source datasets used to derive covariates fulfil this criteria and have a spatial resolution of 100 metres or less. The remaining five source datasets with lower resolutions are included because of their recognized importance for population modelling. The relatively invariable nature of some of these datasets makes their lower resolution less impactful. For example, temperature data, characterized by gradual changes across neighbouring grid cells, remains highly relevant despite its approximately 900m resolution, as sourced from Terra MODIS LST. ^40^

The temporal resolution of source datasets is designed to align with the desired output resolution, ideally covering each year from 2015 to at least 2020, and extending to as recent as 2023 where available. Of the source datasets used, five are time series while 11 are time-invariant. For covariates that typically change slowly over time, such as topography, a single time point is sufficient. In such cases, a time point within the period of interest (2015–2023) is prioritized, with preference given to the most recent data.

Table 1 provides an overview of all source datasets, categorised into one of eight categories. In the following sections, the characteristics of each source dataset are described. In some categories, more than one source dataset is included to provide alternatives for population modelling and other applications or because multiple datasets are combined to calculate new covariates. All data sources are available with the spatial reference GCS WGS 1984.

1. Inland water mask
  Inland water is calculated from the water class ‘Permanent water bodies’ from the land cover classification dataset ESA WorldCover 10m. ^41^ The data product is the first to provide near-global high resolution (10m) land cover classification validated with a global overall accuracy of about 75%. ^42^ ESA WorldCover 10m covers continental land surfaces between latitudes 56S and 82.8N plus European islands and all islands greater than 100 km ^2^ for the years 2020 and 2021. Only 2021 is used to produce the inland water mask.
  The land cover classification is based on Sentinel-1 radar and Sentinel-2 multi-spectral imagery data.
2. Topography
  Topography data consist of MERIT DEM (Multi-Error-Removed Improved-Terrain Digital Elevation Model), ^43^ which is produced by combining two existing baseline DEMs: 1) SRTM (Shuttle Radar Topography Mission) 3 DEM v2.1 ^44^ from the year 2000 with a 100 metres (3 arc-seconds) resolution used for latitudes below 60°N, and 2) AW3D (ALOS: Advanced Land Observing Satellite, World 3D) DEM at 30 metres (1 arc-second) resolution v1 ^45^ from the years 2006-2011 used for latitudes above 60°N. Both of these DEMs have observation gaps, which were filled with Viewfinder Panoramas’ DEM, ^46^ a global void filled DEM of the year 2000 in 100 metres (3 arc-seconds) resolution. Additional supplementary data were then incorporated to correct for the four major error components in spaceborne DEMs – stripe noise, absolute bias, tree height bias, and speckle noise – which particularly improves topography in flat regions like most of the world’s major floodplains and swamp forests. ^43^ MERIT DEM provides a globally consistent terrain elevation dataset covering land areas between 90N and 60S and is supplied as 5 degree Geotiff tiles.
3. Climate
  Climatic variables are obtained from two separate data sources. Precipitation data are taken from TerraClimate, ^47^ a data provider which takes the high spatial resolution (~1km) WorldClim ^48^ dataset and improves its low temporal resolution by using climatic aided interpolation with other data to produce global monthly climatic variables. TerraClimate is distributed as netCDF files containing monthly precipitation bands for the years 1958-2023 at approximately 4km (1/24°) resolution.
  Temperature data consist of the average daytime land surface temperature (LST) variable from the Terra MODIS (Moderate Resolution Imaging Spectroradiometer) Collection 6.1 ^40^ downloaded from Google Earth Engine. The Terra MODIS LST dataset is a monthly composite of temperature given in Kelvin, the composites are based on averaging all cloud free acquisitions from an 8-day-period and supplied as two tiles per year in a netCDF format. ^49^
4. Nighttime lights
  Nighttime lights utilise the annual average masked VIIRS (Visible Infrared Imaging Radiometer Suite) NTL (Night Time Lights) v2.1 and v2.2 data ^50^ from the Earth Observation Group, Payne Institute for Public Policy, Colorado School of Mines. This product offers a consistently processed time series of annual global nighttime lights generated from monthly cloud-free average radiance data, ^50^ with outputs from 2012 to 2023 across both release versions (2012-2021 v2.1 and 2022-2023 v2.2). The masked annual average product provides a data with additional filtering applied; where background noise, in addition to unwanted values from biomass burning or aurora effects, are zeroed out. ^50^ The product has a high spatial resolution of ~500m at the equator and high spatial coverage spanning from 75N to 65S.
5. Land cover
  The above mentioned ESA WorldCover 10m land cover product used to calculate inland water, was not included as the data source for land cover due to its insufficient temporal coverage. Land cover datasets instead consist of ESA (European Space Agency) CCI (Climate Change Initiative) Land Cover (LC) products. The land cover classification gridded maps provide an annual consistent classification of the Earth’s surface into 22 classes from 1992 to 2015 (v.2.0.7cds) and from 2016 to 2022 (v.2.1.1). ^51^ Both versions were produced with the same processing chain and can be used as coherent time series providing full coverage of the period of interest from 2015 to 2022. The ESA CCI LC products have a, for the intended application, suboptimal spatial resolution of approximately 300m. However, the product is still the best choice here as it offers the benefit of consistent classification across the period of interest. In the future it will likely be replaced by a new land cover series Copernicus Global Land Service with a higher resolution of 100 metres ^52^ but with a currently insufficient temporal coverage of 2015-2019. ^53^
6. Protected areas
  The World Database on Protected Areas (WDPA) ^36^ is a comprehensive vector dataset of marine and terrestrial protected areas managed by the UN Environment Programme World Conservation Monitoring Centre (UNEP-WCMC) in collaboration with governments, non-governmental organisations, academia and industry. In order for a protected area to be included in the database it has to meet either the IUCN (International Union for Conservation of Nature) or the CBD (Convention on Biological Diversity) definition. The majority of protected areas, around 90%, represent the boundary of the protected area as polygon data, and only where this is unavailable (multi-)point data are used instead. ^54^ The database is in ESRI file geodatabase format.
7. Infrastructure
  OSM ^37^ data is the largest global and openly accessible database of geographic features, collected by volunteers performing ground surveys to map physical features like roads and buildings and attaching tags for attribution. ^55^ Out of copyright maps and aerial images are also used to add to the data collection ^56^ as are machine learning methods, e.g. to add missing street sections ^57^ or to auto tag in order to aid attribution. ^58, 59^ Due to the multitude of contributors and data sources, completeness and accuracy of OSM data vary. Barrington-Leigh and Millard-Ball ^60^ estimate OSM global road features to be more than 80% complete but highlight considerable heterogeneity in estimated completeness across and within countries. Lloyd, et al. ^32^ suggest that the effective spatial resolution of specifically OSM road data is comparable to SRTM1 (i.e. ~30m at the Equator) but that this varies according to source data. OSM raw data features related to roads and waterbodies are downloaded from Geofabrik ( https://download.geofabrik.de) and supplied as one.osm.pbf file per world sub region (Africa, Antarctica, Asia, Australia and Oceania, Central America, Europe, North America, South America). The MD5 sums for each sub region download are available in Appendix 1.
  Microsoft RoadDetections, ^61^ for simplicity often just called Microsoft Roads, is a second road dataset used to complement OSM data. Microsoft RoadDetections is a near global dataset of 48.9 million km of roads mined from satellite imagery. A Convolutional Neural Network algorithm is trained on 20,000 labelled images with a 1m spatial resolution. It then automatically extracts road pixels from Microsoft Bing Imagery – a composite of several sources – from the years 2020 to 2022. ^61^ In addition Maxar and Airbus imagery are used to supplement the imagery base. Following road pixel extraction, multiple processes are carried out to ensure correct road geometry, this includes for example algorithms for thinning of roads and improvements to road connectivity. ^61^ The dataset is purely spatial, i.e. does not give information about road attributes. As a vector dataset there is no inherent spatial resolution to Microsoft Roads, however because the dataset was produced to complement OSM roads one can assume that it is similar if not higher than that of OSM data. Microsoft Roads can be downloaded as 18 separate .tsv files typically covering a sub-region. The.tsv files contain individual lines of geojson strings describing one road each.
8. Built-up area
  In recent years, datasets describing the built-up environment have received a lot of attention with multiple new geospatial datasets being developed. Datasets can be broadly categorised into ‘building footprints’, which capture the fine-scale geometry of building outlines, and gridded representations of settlements and their characteristics like presence, area or function of built structures. Initial comparisons across some of these datasets have shown that considerable variability exists, ^62, 63^ which may be explained by the difference in underlying satellite imagery, different acquisition times and processing methods. For this category we therefore include seven different source datasets. Some of these datasets, like the building footprint datasets from Microsoft and Google, are used by themselves to directly derive covariates, however, source datasets are also used in combination as detailed in section Spatio-temporal Harmonisation of Covariates, 8. Built-up Area below and Figure 2. The approach for combining multiple datasets is to use the Global Human Settlement Layer (GHSL) P2023 data package ^64^ as the foundation - owing to its comprehensive spatial and temporal coverage - while integrating additional single time point building footprint datasets, as well as the World Settlement Footprint (WSF) 2019 dataset. ^65^ The latter was chosen due to it being generated from optical data combined with radar imagery (Sentinel-1), which is valuable for detecting settlements in areas obscured by cloud cover. By combining datasets in this way, we aim to construct a comprehensive, global multi-temporal analysis of the built-up environment to support improved global multi-temporal population modelling.

**Table 1.** Source datasets used to derive covariates. All datasets have the spatial reference GCS WGS 84.

Category	Name	Aquisition year	Temporal variation	Source	Version, publication year	Data type	Spatial resolution	Format/Pixel type & depth	Spatial coverage
Inland Water Mask	ESA WorldCover 10m	2021	Time Invariant	ESA (European Space Agency) Zanaga, D. et al., 2022	v200	Annual land cover, categorical rasters	10m	Geo-tiff	Global
Topography	MERIT DEM	2000 (SRTM) 2006-2011 (AW3 DEM)	Time invariant	Yamazaki, D. et al., 2017	v1.0.3	Elevation, continuous raster	Typically 3" (~100m)	Geotiff tiles/Float32	Global
Climate	TerraClimate	1958-2023	Time invariant	Abatzoglou, J.T. et al., 2018	2018 (updated 15/04/2021)	Monthly precipitation rasters	1/24° (~4km)	NetCDF; Float32	Global
	Terra MODIS LST (MOD21C3)	2015-2023	Time invariant	Hulley & Hook, 2021	v6.1	Monthly temperature rasters	~1km	NetCDF; Float64	Global
Nighttime Lights	VIIRS NTL	2015-2021 2022-2023	Time Series	Elvidge, C.D. et al., 2021	v2.1 v2.2	Annual average radiance rasters, continuous rasters	15" (~500m)	Geo-tiff/flt32	Between latitudes 75° North and 65° South
Land Cover	ESA CCI Land Cover	2015 2016-2022	Time Series	ESA (European Space Agency) CCI (Climate Change Initiative) - LC (Land Cover Project) Defourny, P. et al., 2019	v2.0.7, 2017 v2.1.1	Annual land cover, categorical rasters	9" (~300m)	Geo-tiff/uint8 netCDF-4	Global
Protected Areas	World Database of Protected Areas (WDPA)	2015-2022	Time Series	UNEP-WCMC & IUCN, 2023	Jan 2023	Terrestrial and marine protected areas, categorical vector	Comparable to 30" (~1km)	ESRI geodatabase	Global
Infrastructure	OpenStreetMap (OSM)	2023	Time invariant	OpenStreetMapFoundation (OSMF) & Contributors, 2023	Jan 2023	General mapping, categorical vector (major raods, waterways and waterbodies)	Comparable to 1" (~30m)	PBF database	Global
	Microsoft RoadDetections	2020-2022	Time invariant	Microsoft, 2023a	2023	Road lines, vector	Comparable to 1" (~30m)	TSV of GeoJson	Global
Built-up Area	Global Human Settlement Layer (GHSL) BUILT-S	2015, 2020, 2025, 2030	Time Series	Pesaresi, M. and Politis, P., 2023a	GHSL Data Package 2023	Built-up surface, continuous raster	100m	Geo-tiff, uint16	Global
	Global Human Settlement Layer (GHSL) BUILT-V	2015, 2020, 2025, 2030	Time Series	Pesaresi, M. and Politis, P., 2023b	GHSL Data Package 2023	Built-up volume, continuous raster	100m	Geo-tiff, uint32	Global
	Global Human Settlement Layer (GHSL) BUILT-H	2018	Time invariant	Pesaresi, M. and Politis, P., 2023c	GHSL Data Package 2023	Built-up height, continuous raster	100m	Geo-tiff, uint32	Global
	Microsoft Building Footprints	2014-2021	Time invariant	Microsoft, 2023b	2023	Building footprint, vector	0.3m (underlying imagery)	TSV of GeoJson	Global
	Google Open Buildings	pre 2021	Time invariant	Sirko, S. et al., 2021	v3	Building footprint, vector	0.5m (underlying imagery)	CSV of GeoJson	Global
	World Settlement Footprint (WSF) 2019	2019	Time invariant	German Aerospace Center (DLR), 2021 Marconcini, M. et al., 2021	2021	Settlement mask, binary raster	10m	Geo-tiff	Global
	Open Street Map (OSM)	2023	Time Invariant	Open Street Map Foundation (OSMF) & Contributors, 2023	Jan 2023	General mapping, categorical vector (non-residential buildings)	Comparable to 1" (~30m)	PBF database	Global

From the GHSL P2023 data package the datasets GHSL BUILT-S, ^66^ GHSL BUILT-V ^67^ and GHSL BUILT-H ^68^ are used. GHSL BUILT-S depicts the built-up surface in square meters delineated into total built-up surface and only non-residential built-up surface. In this context, built-up surface can be understood as any above ground structure with a roof. ^64^ GHSL BUILT-V depicts the built-up volume expressed as number of cubic meters and is also stratified into total built-up volume and non-residential built-up volume. GHSL BUILT-S and GHSL BUILT-V are raster datasets available in 5 year intervals starting in 1975. To cover the study period the years 2015, 2020, 2025, and 2030 are used with the future years 2025 and 2030 being produced by spatial-temporal extrapolation. ^64^ These data are largely derived from Sentinel-2 composites and Landsat data, methodologies are detailed in Pesaresi, et al. ^69^ and the GHSL P2023 release document. ^64^ GHSL BUILT-H describes the spatial distribution of average building heights for the year 2018. The GHSL P2023 data package has a global coverage with a 100 metres resolution.

The World Settlement Footprints (WSF) 2019 dataset ^65^ provided by the German Aerospace Centre (DLR), is another gridded settlement dataset utilised. This high-resolution (10 metres) dataset offers a binary mask depicting the global distribution of human settlements for the year 2019. It was derived by combining multitemporal Sentinel-1 radar and Sentinel-2 multi-spectral satellite imagery. ^70^ A random forest model trained on settled versus non-settled sample points in 30 different climate types performs the classification which is followed by masking out of roads. ^70^

The built-up environment is furthermore described by three polygon datasets of building footprints.

The Google Open Buildings ^71^ dataset provides 1.8 billion building footprints extracted from 50cm resolution satellite imagery for the Global South. Google does not give details on the acquisition years of the imagery but it is likely to be in the recent years before the first data release in 2021. A convolutional neural network is trained to determine segmentation of satellite images in two stages: first each individual pixel is classified as building or non-building; building pixels are subsequently connected based on confidence score thresholds and converted to polygons. ^39, 71^ Final confidence scores are supplied for each individual building. The dataset was initially developed for the continent of Africa but has since been extended to include new regions in South Asia and South East Asia and Latin America and the Caribbean. Coverage of these regions is however not necessarily complete. In Africa, for example, the underlying high resolution imagery covered 64% of the continent’s land surface, concentrating on and capturing the majority of settled areas. ^71^

Microsoft Building Footprints ^38^ is a second vector dataset of over one billion building footprints produced through automated extraction. The underlying satellite imagery with a 30cm resolution is taken between 2014 and 2021 by Bing Maps, Maxar and Airbus and are located around the world. Similar to Google Open Buildings V3 Polygons, Microsoft Building Footprints are also extracted through a deep learning model working in the two stages of semantic segmentation to classify pixels containing buildings using a convolutional neural network with subsequent detection of groups of connected building pixels and polygonization. Confidence scores are supplied only for buildings captured since the December 2022 release. ^38^

Finally, building footprints from OSM raw data are downloaded from Geofabrik ( https://download.geofabrik.de) as.osm.pbf file in a similar manner to the OSM infrastructure data described above.

### Construction of a global master grid

Prior to covariate processing, a base grid used for spatial alignment and resolution is created, henceforth called master grid. The master grid has a resolution of approximately 100 metres at the Equator (3 arc-seconds) and joins up at the datum line, covering all major landmasses between approximately 60° South (59.9999994°S to be precise) and 84° North. Higher latitudes are excluded due to negligible population.

To distinguish between oceans and landmasses, the latter are defined by combining all terrestrial land cover classes (tree cover, shrubland, grassland, cropland, built-up, bare/sparse vegetation, snow and ice, herbaceous wetland, mangroves, moss and lichen) from the ESA WorldCover 10m ^41^ dataset. These classes are aggregated and then resampled to the output resolution. The coastline is defined by a 25% terrestrial share threshold, ie grid cells with at least 25% of their area classed as terrestrial are considered part of the landmass. Oceans and seas, such as the Black Sea or Caspian Sea, are registered as NoData, while rivers, lakes and other inland water bodies are registered as part of the landmass.

The landmass is further split into countries and territories using the Large Scale International Boundaries (LSIB) Dataset v11.3, ^72^ which reflects U.S. Government policy on international boundary alignment, political recognition and dispute status. 41 boundaries are ambiguous and form small areas which are not clearly assigned to a country/territory. In these cases Global Mosaiced National Boundaries produced by WorldPop, ^73^ the GPWv4 National Identifier Grid ^14^ and boundaries related to the latest national census are used as source for delineation of countries/territories. Southern Asia contributed the largest proportion of these small areas by sub-region. Finally, each country/territory is given a unique three digit numerical code in line with ISO-3166. ^74^

### Spatio-temporal harmonisation of covariates

The harmonisation processing for each source dataset into a standardised geospatial covariate layer is described below as well as in Figure 1 and Figure 2.

**Figure 3.**
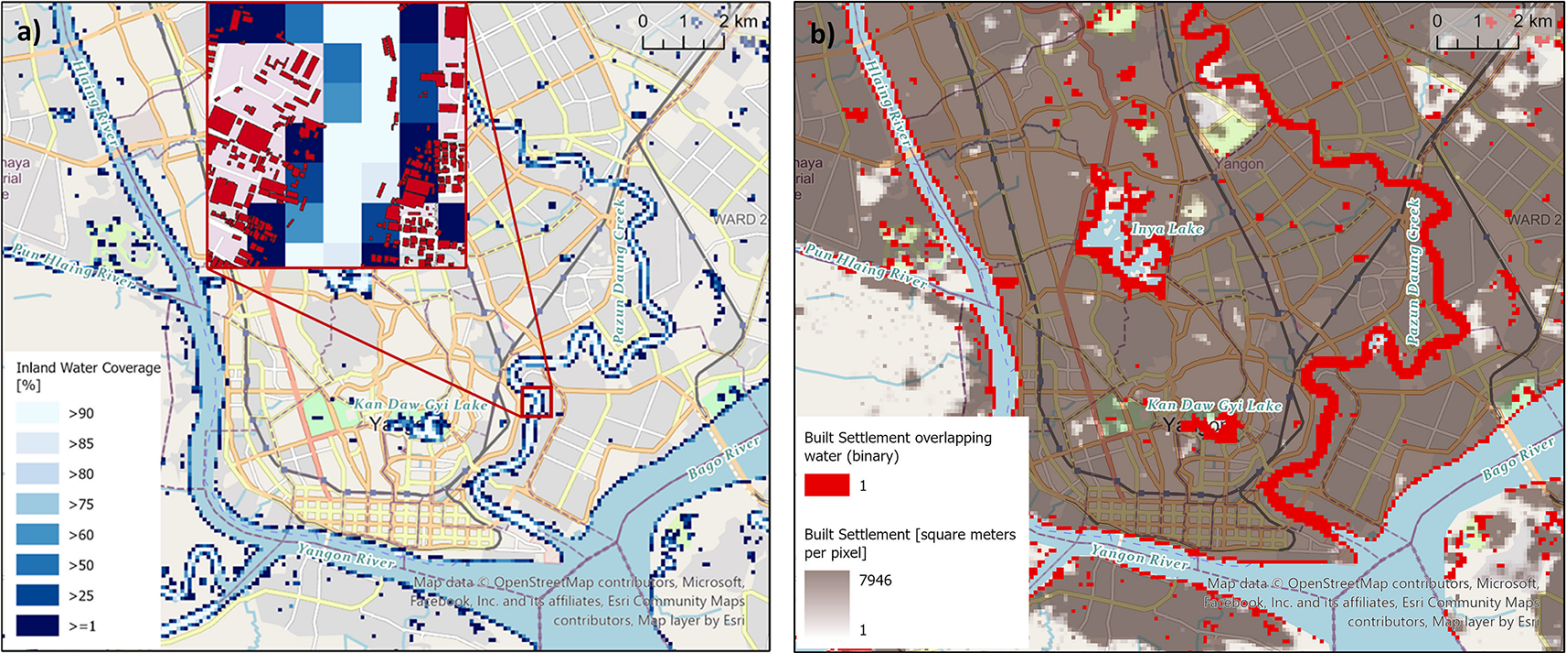
Inland Water Coverage along rivers and lakes in Yangon, the largest city of Myanmar. a) Varying thresholds of inland water share to define the inland water mask (main map) and affected building footprints (inset map); and b) its relevance to built-up areas.

**Figure 4.**
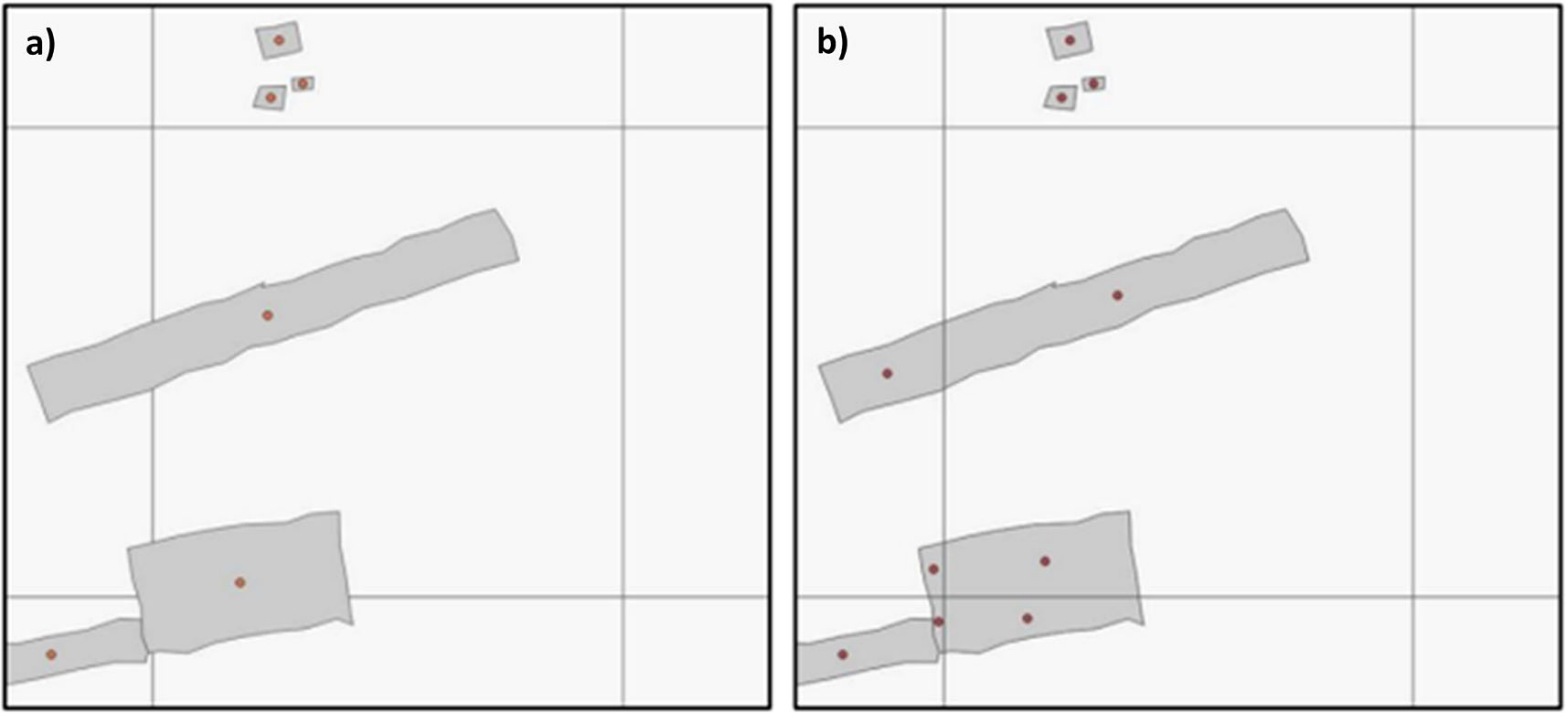
Methods to derive covariates from building footprints. a) The Building Centroid Based (BCB) method allocates values to grid cells based on building centroids located within respective grid cells’ bounds. In cases where buildings straddle multiple grid cells, their metrics (area, length, etc.) will only be allocated to the grid cell in which the buildings’ centroids are located. b) The Pixel intersected Based (PIB) method makes use of the geometric intersections between building footprints and grid cells to calculate values, resulting in building metrics being allocated to all grid cells in which building footprints are present.

1. Inland water masks
  An inland water mask is necessary to avoid the placing of populations inside water bodies. For this purpose ESA WorldCover 10m v200 for 2021 ^41^ is used, in particular class 80 ‘Permanent water bodies’ which includes natural (e.g. lakes, rivers) and artificial (e.g. reservoirs, canals) areas covered by fresh or salt water for most of the year. The processing of ESA WorldCover 10m data requires considerable computational power due to its fine resolution and is carried out on the HPC system. The extracted raster is resampled to match master grid specification. Grid cells related to oceans and coastlines are removed, leaving only inland water bodies with values describing the water share of each pixel. Instead of applying a majority rule to create one binary mask, a set of raster files is calculated representing varying thresholds of inland water coverage per grid cell, specifically thresholds of 25%, 50%, 60%, 75%, 80%, 85% and 90%. This allows users to choose the mask that best fits their application and area of interest. The effect of different thresholds will be largest on lake- or riverside settlements. Figure 3 shows the example of the city Yangon, Myanmar, located at the confluence of the Bago and Hlaing rivers. Figure 3a depicts the grid cells classed as inland water depending on the different thresholds; the lower the chosen threshold, the more grid cells are included in the water mask. The relevance of choosing a context specific threshold becomes clear in Figure 3b, where the bright red colour indicates settled grid cells with an inland water share. For the derived covariate ‘distance to inland water’ a 75% threshold was applied.
  This new water mask resolves previously known issues with the water mask of Lloyd, et al., ^32^ where the application of the mask led to the potentially loss of land pixels due to discrepancies between the mask and coastline.
2. Topography
  MERIT DEM ^43^ five degree tiles are mosaicked into one global layer. Subsequently, the elevation grid is clipped and resampled to the master grid standard and the NoData pixel value adjusted accordingly. Where there are gaps along the coastline due to inconsistencies between the master grid and elevation coastline, they are filled with the neighbouring mean elevation value to provide a complete representation of elevation for all landmasses. Finally, a topographic slope layer is derived. The entire workflow uses GDAL utilities.
3. Climate
  Monthly TerraClimate precipitation data ^47^ is extracted from bands 1-12 of the annual netCDF files to create individual GeoTiffs for each month of each reference year. In the next step, monthly precipitation rasters are summed up according to each reference year and divided by 12 to produce annual average precipitation. The NoData Value is adjusted. As last step the rasters are resampled using a bilinear approach to master grid specification and missing pixels are filled with neighbouring mean values to provide complete coverage.
  Two Terra MODIS LST ^40^ monthly temperature composites with combined global extent are initially aggregated to annual datasets via Google Earth Engine. The remaining workflow is carried out in GDAL where the rasters are mosaicked to one global file for each year and adjusted to match the extent of the master grid. In the same manner as described for the precipitation rasters above, the temperature raster’s NoData value is adjusted, the rasters are resampled and missing pixels filled.
4. Nighttime lights
  The annual average masked VIIRS NTL v2.1 and v2.2 radiance grids are assigned a suitable NoData value, and are directly standardised to master grid extent and resolution using bilinear resampling. NoData pixels between 75N and 84N are set to zero. The annual rasters are adjusted to match the master grid’s coastline, turning all pixels outside of the landmass to NoData.
  Annual average masked VIIRS NTL v2.1 and v2.2 radiance grids are already supplied with a correction for clouds and outliers. Despite the implemented outlier removal, some pixels with abnormally large radiance values, possibly caused by volcanoes or large fires, can be observed. Locations of global gas flaring sites ^75^ and volcanoes ^76^ are used to further filter the abnormal values in the data which are unlikely to appropriately reflect the presence of human settlement. The outlier removal algorithm developed for DMSP-OLS nighttime lights ^77^ has been adopted to suit VIIRS NTL. The algorithm iteratively removes the largest visible band observation, recomputing the standard deviation of the remaining observations, and then compares the new standard deviation to that of the previous iteration. This process stops when the standard deviations converge, which is defined as a difference of less than 0.2. This workflow will be published in detail as a stand alone paper.
5. Land cover
  Following the workflow outlined in Lloyd, et al. ^32^ and carried out with GDAL utilities, ESA CCI Land Cover annual land cover maps are first converted to GeoTIFFs, the original 22 classes are then simplified and aggregated to create nine new classes essentially removing subclasses for greater efficiency in the intended purpose. The separate classes are combined into one annual grid containing all classes which in turn is down sampled using nearest neighbour to match the master grid extent and resolution. Binary files are extracted for each of the nine new classes where a pixel value of 1 represents the land cover class of interest. Gaps along the coastline within these binary files are filled with the most frequent value from the neighbouring grid cells. The final step is to convert the binary categorical rasters to continuous rasters by calculating the nearest distance from each pixel to the land cover class boundary of interest.
6. Protected areas
  The WDPA is imported into PostGIS for ease of processing; quality checks lead to omitting of 13 features due to missing geometries. Following Lloyd, et al., ^32^ protected areas represented by point data are converted to polygons through geodesic buffering with a 70m radius to serve as proxy of the protected area; they are then merged with the original polygon data to create one single dataset. The protected areas polygon dataset is further manipulated to contain integer values which facilitate SQL queries to separate marine protected areas from terrestrial protected areas and to distinguish between IUCN category 1 protected areas (category 1a ‘strict nature reserves’ and category 1b ‘wilderness areas’ ^78^) and other IUCN categories 2 to 6. The remaining workflow is carried out with GDAL utilities. Each category’s dataset is incrementally rasterized to form an annual time series from the year 2015 to 2022 of global GeoTiff mosaics of protected area locations matching master grid resolution and alignment. Each year refers to protected areas registered in that year and earlier, i.e. the dataset for 2015 contains protected areas registered from 1819 (the year the first record dates back to) up to and including 2015. Each of the 16 terrestrial mosaics (eight annual mosaics for IUCN category 1 plus eight annual mosaics for IUCN categories 2 to 6) is clipped to remove protected areas outside the landmass depicted by the master grid and merged with the corresponding marine dataset of the same year. This is done because marine protected areas are not strictly constrained to aquatic environments but can straddle into terrestrial terrain along coastlines. The mosaics are then clipped once more to remove protected areas falling outside the landmass depicted by the master grid. The result is two mosaics per year, one for IUCN category 1 protected areas (strict nature reserve and wilderness areas), and one for IUCN categories 2-6 protected areas (national park, national monument, habitat species management, protected landscape/seascape and managed resource protected areas). Finally, these categorical rasters are converted to continuous rasters by calculating the distance to the closest protected area boundary for each grid cell.
7. Infrastructure
  OSM ^37^ data are processed in line with the detailed descriptions given in Lloyd, et al. ^31^ and Lloyd, et al. ^32^ The process can be summarised as extraction of features of interest and mosaicking into three separate layers. One layer is created for major highways representing road priorities from tertiary roads up to motorways, one layer for major road intersections and a third one for major natural waterways. The individual layers are rasterised and standardised to master grid specification before distances to OSM features are calculated from the cell centres to the nearest feature.
  Processing of Microsoft Roads ^61^ first includes the conversion of individual road .tsv files to the GeoPackage format for easier handling, followed by deriving three covariates based on the vector geometries directly: count of roads per grid cell, total length of roads in metres per grid cell, and road density by dividing the total road length per pixel by the area of the pixel. In addition, roads are rasterised to master grid specification to form a binary representation of presence of roads. The last derived covariate calculated from this source dataset is the distance from each grid cell to the nearest road.
8. Built-up area
  Individual covariates are derived directly from the two building footprint datasets, Google Open Buildings ^39^ and Microsoft Building Footprints ^38^ with identical processing steps performed on the HPC utilising building footprints of all confidence levels. Two different methods are used to derive covariates from the footprints, see Figure 4. The Building Centroid Based (BCB) method allocates values to grid cells based on building centroids located within respective grid cells’ bounds. In cases where buildings straddle multiple grid cells, their metrics (area, length, etc.) will only be allocated to the grid cell in which the buildings’ centroids are located. This method is more suited to building count and distance-to-neighbour related metrics. The second method - Pixel Intersected Based (PIB) method - makes use of the geometric intersections between building footprints and grid cells to calculate values, resulting in building metrics being allocated to all grid cells in which building footprints are present. This method results in a more continuous grid, with exact measurements related to building area and length being allocated to the pixels in which they are proportionally located. Based on those two approaches the building characteristics count, total area, mean area, and variation coefficient of area, total perimeter length, mean perimeter length, and variation coefficient of perimeter length as well as building density are calculated. Building density is defined as the count of buildings within grid cells divided by the grid cell’s area.
  The total built-up area and non-residential built-up area are further described through the combination of multiple settlement and building footprint datasets as shown in Figure 2.

**Figure 5.**
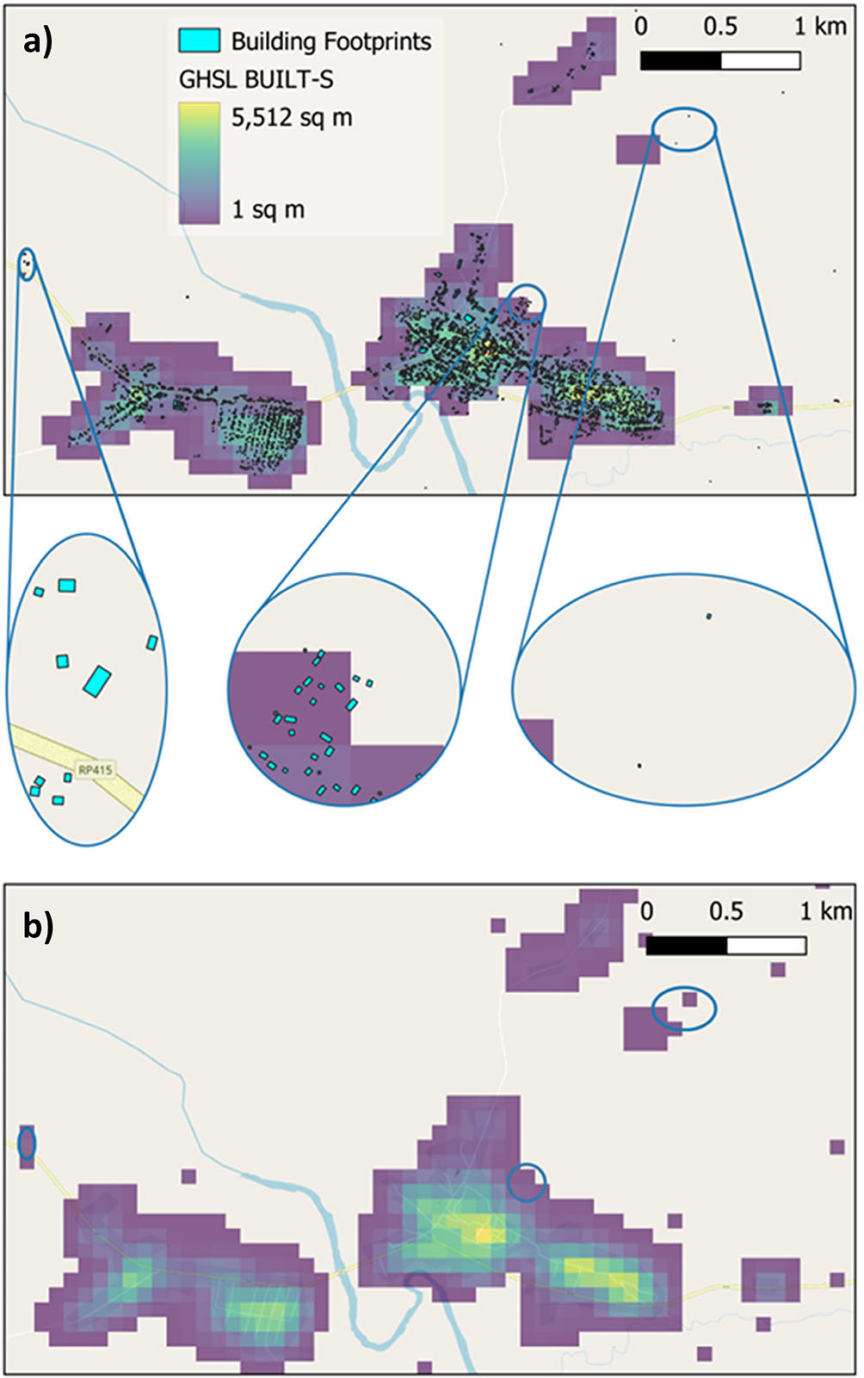
Example of combination of GHSL BUILT-S (surface) data and building footprint datasets to produce covariates around the built-up environment. a) The GHSL BUILT-S grid forms the basis for the built-up area. Overlaying building footprints from Google Open Buildings and Microsoft building footprints shows that these datasets identify additional buildings. In this example of the area around the settlement Zobia in the Democratic Republic of the Congo one additional hamlet, urban expansion and multiple isolated buildings are visible (elliptical insets maps from left to right). b) The surface area of these additional building footprints has been calculated and the relevant grid cells added to GHSL BUILT-S to produce a more comprehensive built-up area.

GHSL BUILT-S ^66^ (surface) forms the basis for the total built-up area (residential plus non-residential). It was chosen for its comprehensive spatial coverage and multi-temporal nature. As it is supplied as raster data, it is resampled directly to match master grid specifications and then modified through addition of the single time point datasets Google Open Buildings, Microsoft Building Footprints, and WSF 2019. An example is shown in Figure 5. The following logic is used: Where built-up grid cells in GHSL BUILT-S for year 2030 are missing but exist in any of the other building footprint or settlement datasets, the total built-up area is added to the GHSL BUILT-S layer for all years, i.e. 2015, 2020, 2025 and 2030 (see Figure 2a). The same process is applied to GHSL BUILT-V ^67^ (volume) data. As neither the building footprint datasets (Google Open Building Footprints, Microsoft Building Footprints) nor the settlement dataset (WSF 2019) contain information about building height, each building footprint/settled grid cell absent in GHSL BUILT-V is assigned an estimated height based on Nadaraya–Watson kernel regression ^79^ informed from nearby built-up grid cells existing in GHSL BUILT-H. ^68^ The estimated height is then multiplied by the known surface area to derive volume, which in turn is added to GHSL BUILT-V ( Figure 2c).

Another set of covariates specifically for non-residential built-up areas is produced. GHSL provides layers for non-residential built-up surfaces and non-residential built-up volume, whereas building footprints from Google and Microsoft do not distinguish between residential and non-residential use. Similarly, WSF 2019 does not provide this distinction. Therefore building footprints from OSM with building type tags indicating non-residential use – for example ‘public’, ‘industrial’ or ‘outbuilding’ (see Supplementary Information for the complete list) - are incorporated instead. With the logic described above, GHSL BUILT-S and GHSL BUILT-V layers for non-residential buildings only are modified through the addition of total area and total volume respectively of non-residential buildings from OSM ( Figure 2b and Figure 2d).

## Data records

**Figure 6.**
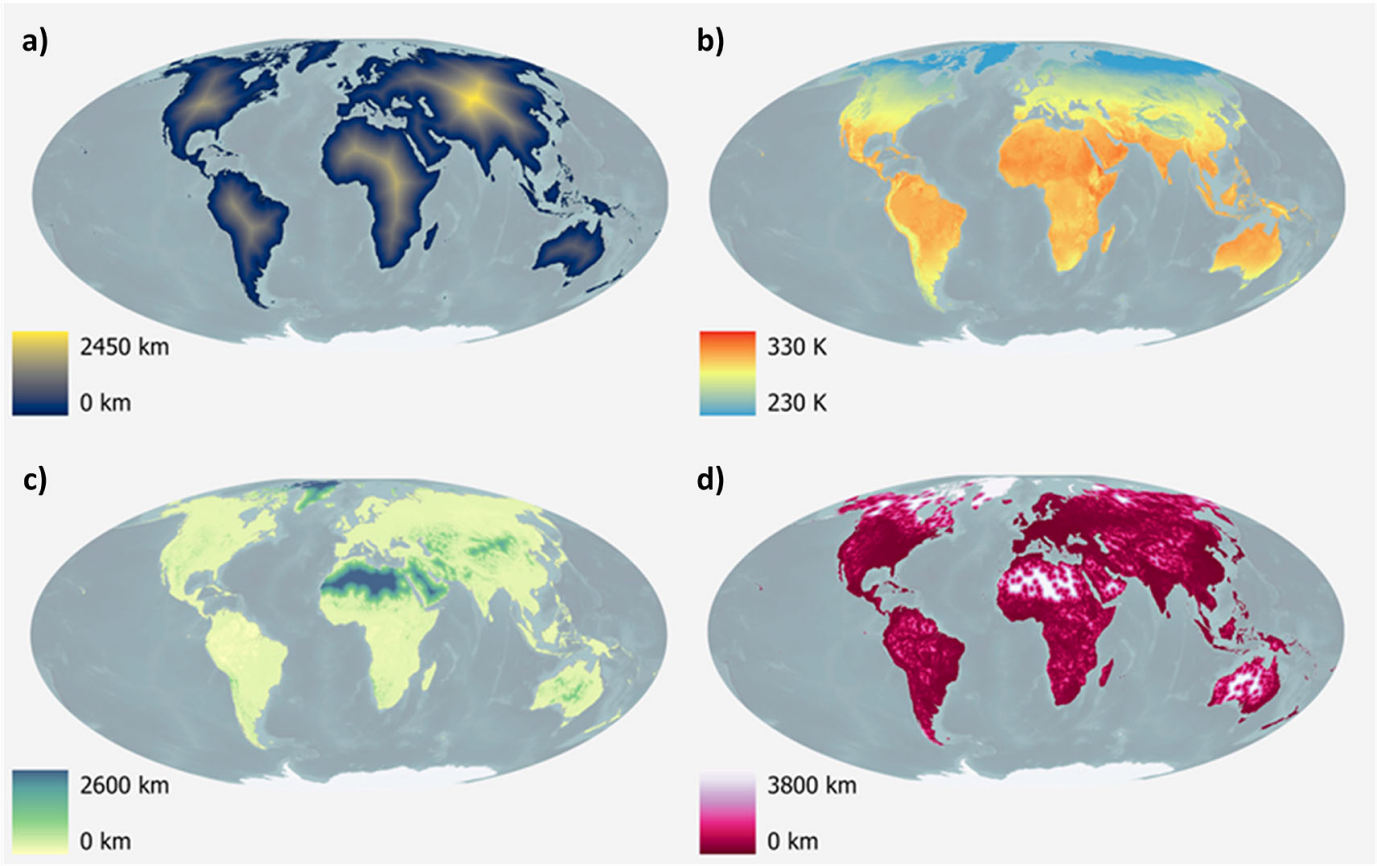
Global visualisation of selected covariates. a) Distance to open-water coastline, b) Annual mean surface temperature for the year 2023, c) Distance to woody-tree area edges for the year 2022, d) Distance to artificial surface area edges for the year 2022.

A selection of covariates is visualised in Figure 6. The complete collection of 73 geospatial covariates is depicted in Table 2.

**Table 2.** Summary of Covariates produced including their source dataset and temporal coverage.

Category	Source dataset name	Temporal coverage of covariate(s)	Covariate(s)
Inland Water Mask	ESA WorldCover 10m v200	2021	Inland water coverage [%]
			Presence of inland water based on different thresholds of water coverage (25%, 50%, 60%, 75%, 80%, 85% and 90%) [binary]
			Distance to inland water [km]
			Distance to open-water coastline [km]
Topography	MERIT DEM	~2007	MERIT-based Elevation [m]
			MERIT-based Slope [°]
Climate	Terra MODIS LST	2015-2023 (time series)	Annual Mean Surface Temperature [Kelvin]
	TerraClimate	2015-2023 (time series)	Annual Mean Precipitation (scaling factor 10] [mm]
Nighttime Lights	VIIRS NTL 2.1	2015-2021 (time series)	Night time lights, flares/volcanoes filtered [nW/cm2/sr]
	VIIRS NTL 2.2	2022-2023 (time series)	Night time lights, not flares/volcanoes filtered [nW/cm2/sr]
Land Cover			Distance to cultivated area edges (reclassified class 11) [km]
			Distance to woody-tree area edges (reclassified class 40) [km]
			Distance to shrub area edges (reclassified class 130) [km]
	ESA CCI Land Cover v2.0.7	2015	Distance to herbaceous area edges (reclassified class 140) [km]
			Distance to sparse vegetation area edge (reclassified class 150) [km]
	ESA CCI Land Cover v2.1.1	2016-2022 (time series)	Distance to aquatic vegetation area edges (reclassified class 160) [km]
			Distance to artificial surface area edges (reclassified class 190) [km]
			Distance to bare area edges (reclassified class 200) [km]
			Distance to Water, Snow and Ice (reclassified class 210) [km]
Protected Areas	World Database of Protected Areas (WDPA)	2015-2022 (time series)	Distance to IUCN strict nature reserve and wilderness area edges [km]
			Distances to IUCN national park, national monument, habitat species management, protected landscape/seascape and managed resource protected area edges [km]
Infrastructure	OpenStreetMap (OSM)	2023	Distance to OSM major roads [km]
			Distance to OSM major road intersections [km]
			Distance to OSM waterbodies [km]
	Microsoft RoadsDetection	~2021	Count of Microsoft Roads
			Presence of Microsoft Roads [binary]
			Microsoft Roads Density
			Distance to Microsoft Roads [km]
			Total length of Microsoft Roads [m]
Built-up Area	Microsoft Building Footprints	~2000	Count of MS Buildings ( *PIB/BCP)
			Variation coefficient of MS Buildings’ area ( *PIB/BCP)
			MS Building density ( *PIB/BCP)
			Total area of MS Buildings ( *PIB/BCP)
			Mean area of MS Buildings ( *PIB/BCP)
			Total length of perimeter of MS Buildings ( *PIB/BCP)
			Mean length of perimeter of MS Buildings ( *PIB/BCP)
			Variation coefficient of MS Buildings’ perimeter length ( *PIB/BCP)
	Google Open Buildings Footprints	~2000	Count of Google Buildings ( *PIB/BCP)
			Google Building density ( *PIB/BCP)
			Total area of Google Buildings ( *PIB/BCP)
			Mean area of Google MS Buildings ( *PIB/BCP)
			Total length of perimeter of Google Buildings ( *PIB/BCP)
			Mean length of perimeter of Google Buildings ( *PIB/BCP)
			Variation coefficient of Google Buildings’ area ( *PIB/BCP)
			Variation coefficient of Google Buildings’ perimeter length ( *PIB/BCP)
	Global Human Settlement Layer (GHSL) BUILT-S	2015, 2020, 2025, 2030	Built-up Surface ** [m ^2^]
			Non-residential Built-up Surface *** [m ^2^]
			Distance to Built-up Surface ** [km]
			Presence of Built-up Surface ** [binary]
	JRC Global Human Settlement Layer (GHSL) BUILT-V	2015, 2020, 2025, 2030	Built-up Volume **** [m ^3^]
			Non-residential Built-up Volume ***** [ m ^3^]

* Building metrics are calculated based on the pixel in which their respective centroids are located (BCB method), or the pixel(s) that their geometries intersect (Pixel intersected Based – PIB method).

** GHSL BUILT-S data are combined with Microsoft Building Footprints, Google Open Buildings Footprint and with World Settlement Footprint. Missing built-up pixels in GHSL but existing built-up pixels in Microsoft Building footprints, Google Building footprints and/or World Settlement Footprint were added to the base GHSL layer.

*** Non-residential GHSL BUILT-S data are combined with non-residential building footprints from OpenSreetMap. Missing built-up pixels in GHSL but existing built-up pixels in OpenSreetMap were added to the base GHSL layer.

**** GHSL BUILT-V data are combined with Microsoft Building Footprints, Google Open Buildings Footprint and with World Settlement Footprint. Missing built-up pixels in GHSL but existing built-up pixels in Microsoft Building footprints, Google Building footprints and/or World Settlement Footprint were transformed to building volumes through the help of interpolated GHSL BUILT-H data and added to the base GHSL layer.

***** Non-residential GHSL BUILT-V data are combined with non-residential building footprints from OpenSreetMap. Missing built-up pixels in GHSL but existing built-up pixels in OpenSreetMap were transformed to building volumes through the help of interpolated GHSL BUILT-H data and added to the base GHSL layer.

The covariates are organised at the country level, for 242 countries in total, and are publicly available in GeoTIFF format. All gridded datasets have a spatial resolution of 100 metres at the equator (3 arc-seconds) with the spatial reference WGS84 and a NoData value of -99999.

The section Data Availability gives details how to access the covariates and provides a recommended citation.

## Technical validation

The collection of covariates presented here is derived through commonly used geoprocessing workflows to spatio-temporally harmonise all covariates to the same grid specifications. The technical quality of the covariates is therefore largely dependent on the quality of the source dataset they are derived from, which are validated and discussed by independent publications topography, ^43, 80^ protected areas, ^81– 85^ climate ^86– 93^; land cover, ^42, 94, 95^ building footprints ^62, 63, 96, 97^ and built-up areas, ^62, 71^ infrastructure. ^98– 101^ Where some countries/territories are missing in the Microsoft RoadDetections dataset, the corresponding covariates are still included in this publication but will show zeros. Differences in country boundaries can lead to cases where a country for which data were originally not published, receives some roads or roads segments of neighbouring countries. Appendix 3 gives an overview of percentage of country covered by Microsoft Roads. Users are urged to assess coverage of their country/territory of interest before using this covariate.

Quality assessments during the harmonisation process itself were carried out diligently and included technical checks around extent, number of rows and columns, resolution, spatial reference and NoData values. Visual checks were also carried out for absence of abnormal areas of NoData and to ensure data continuity across the datum line. When splitting global covariates into individual countries/territories the extent and number of total valid pixels were compared across covariates (see Supplementary Information).

## Usage notes

**Figure 7.**
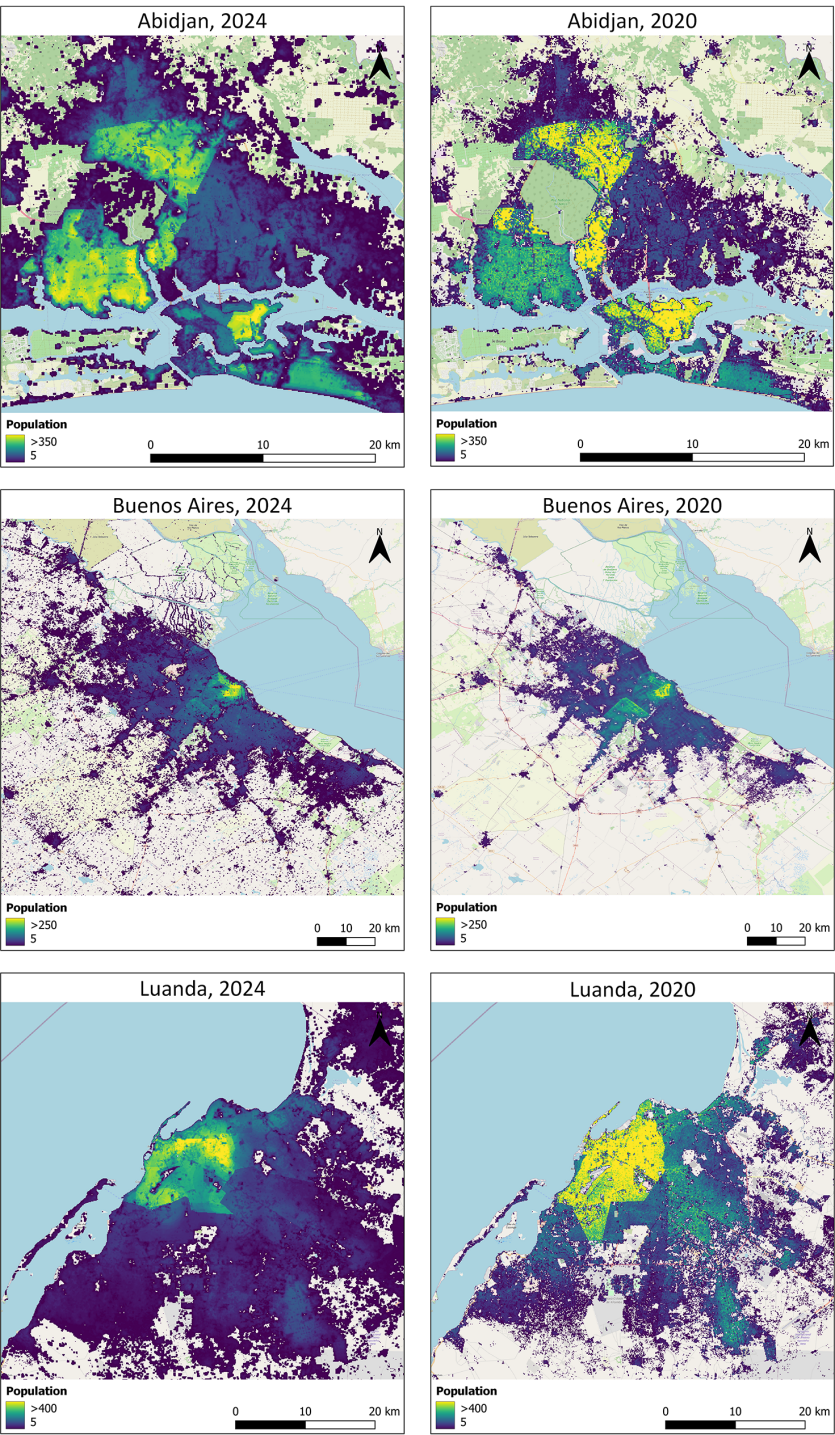
Comparison of population count estimates modelled from the here presented covariates (year 2024) and population count estimates modelled from covariates by Lloyd et al. 2019 (year 2020) for Abidjan (Ivory Coast), Buenos Aires (Argentina) and Luanda (Angola). The increase in populated areas seen are partly due to urban infill and expansion and also due to the change in covariates, in particular covariates describing the built-up area. To facilitate comparison of the effect of the change in covariates, the same modelling technique was used and the underlying administrative units have remained stable between the two years.

The collection of consistent, standardised, global covariates was predominantly produced under the premise of enabling the wider research community to apply the covariates in statistical models - like the Random Forest model ^20^ - to inform global, high resolution population distribution mapping. Figure 7 presents a comparison between population estimates derived from the covariates introduced in this study and those generated using the covariates from Lloyd, et al. ^32^ However, individual or multiple covariate datasets can also support and broaden applications across environmental, economic, and health sectors.

The master grid presented here differs compared to previous iterations of global covariates and population datasets produced by WorldPop. Long-term users of WorldPop’s global data products need to be aware that grid cells do not align to previous data, equally boundaries and extents of individual countries/territories can be different.

Users are urged to choose covariates regarding the built-up environment carefully and note the difference between the PIB and BCB method as explained above. It should be noted that the number of valid (non-NoData) grid cells in the PIB method will most likely be greater than the corresponding dataset using the BCB method, due to the BCB method only considering building centroids in its calculations. Users need to read the descriptions given in the Methods section and metadata to select the most appropriate dataset for their specific requirements. For some themes multiple covariates from different data sources were produced, one example are building footprints from Google and Microsoft. Gonzales ^62^ initial comparison of these showed that both datasets perform similarly in rural areas, while Microsoft generates fewer but larger building footprints in an urban context. Chamberlain, et al. ^63^ found large variations between building footprints products and noted the generally lower values in terms of total building area and building counts of Microsoft building footprints. Users are therefore advised to carefully evaluate which dataset best meets their needs. Users are also made aware that the LSIB dataset used to define country and territory boundaries reflect U.S. Government policy on international boundary alignment, political recognition, and dispute status. They do not necessarily reflect de facto limits of control and neither the U.S. Government nor the authors of this manuscript give any warranties as to the validity, accuracy, completeness, or fitness for a particular purpose. Some very small land areas are left unassigned from the LSIB dataset and covariates are not being produced for these.

Future versions of the collection of covariates are likely to be released as source datasets for recent years and additional source datasets become available; one example is the addition of a higher resolution land cover dataset (LandCover CGLS-100m ^52^) once updates from Sentinel data are available to form a complete time series. A separate publication about the precise methodology used to filter out fires and volcanoes from the VIIRS nighttime lights data is also intended.

## Code availability

The code used for the harmonisation of covariates is available via the GitHub repository https://github.com/wpgp/global2_scripts (doi: 10.5281/zenodo.14236240). The code may be distributed using a Creative Commons Attribution 4.0 License.

## Data Availability

The complete collection of 73 geospatial covariates is publicly available in GeoTIFF format via the WorldPop hub https://hub.worldpop.org/project/categories?id=14. The hub is recommended for users interested in downloading individual covariate datasets. For bulk access, the complete collection of covariates for each country can be downloaded via the WorldPop FTP server ftp://ftp.worldpop.org/GIS/Covariates/Global_2015_2030 , which provides a streamlined option for retrieving all datasets in a single operation. Covariates are available under the terms of the Creative Commons License (CC BY 4.0), with the exception of datasets derived from OpenStreetMap, Microsoft Building Footprints or Microsoft RoadsDetection, which are available under the Open Database License (ODbL). The recommended data citation for the covariate collection is: *D. Woods, T. McKeen, A. Cunningham, R. Priyatikanto, A. Sorichetta, A.J. Tatem and M. Bondarenko. 2024. WorldPop high resolution, harmonised annual global geospatial covariates. Version 1.0. University of Southampton: Southampton, UK. DOI:10.5258/SOTON/WP00772* Supplementary Information includes four appendices (Appendix 1: OSM MD5 sums per sub region download; Appendix 2: OSM tags to define non-residential buildings; Appendix 3: Microsoft Roads Detection Coverage by Region; Appendix 4: Number of total valid pixels per country/territory), which are available via the Zenodo repository under the terms of the Creative Commons License (CC BY 4.0), https://doi.org/10.5281/zenodo.15525473.
